# Temporal gene expression profiling reveals CEBPD as a candidate regulator of brain disease in prosaposin deficient mice

**DOI:** 10.1186/1471-2202-9-76

**Published:** 2008-08-01

**Authors:** Ying Sun, Li Jia, Michael T Williams, Matt Zamzow, Huimin Ran, Brian Quinn, Bruce J Aronow, Charles V Vorhees, David P Witte, Gregory A Grabowski

**Affiliations:** 1 Division of Human Genetics, Cincinnati Children's Hospital Medical Center, 3333 Burnet Avenue, MLC 4006, Cincinnati, OH, USA; 2 Division of Neurology, Cincinnati Children's Hospital Medical Center, Cincinnati, OH, USA; 3 Division of Pediatric Pathology, Cincinnati Children's Hospital Medical Center, Cincinnati, OH, USA; 4 Divisions of Biomedical Informatics and Developmental Biology, Cincinnati Children's Hospital Medical Center, Cincinnati, OH, USA; 5 Department of Pediatrics, University of Cincinnati College of Medicine, Cincinnati, OH, USA; 6 Department of Pathology, University of Cincinnati College of Medicine, Cincinnati, OH, USA

## Abstract

**Background:**

Prosaposin encodes, in tandem, four small acidic activator proteins (saposins) with specificities for glycosphingolipid (GSL) hydrolases in lysosomes. Extensive GSL storage occurs in various central nervous system regions in mammalian prosaposin deficiencies.

**Results:**

Our hypomorphic prosaposin deficient mouse, PS-NA, exhibited 45% WT levels of brain saposins and showed neuropathology that included neuronal GSL storage and Purkinje cell loss. Impairment of neuronal function was observed as early as 6 wks as demonstrated by the narrow bridges tests. Temporal transcriptome microarray analyses of brain tissues were conducted with mRNA from three prosaposin deficient mouse models: PS-NA, prosaposin null (PS-/-) and a V394L/V394L glucocerebrosidase mutation combined with PS-NA (4L/PS-NA). Gene expression alterations in cerebrum and cerebellum were detectable at birth preceding the neuronal deficits. Differentially expressed genes encompassed a broad spectrum of cellular functions. The number of down-regulated genes was constant, but up-regulated gene numbers increased with age. CCAAT/enhancer-binding protein delta (CEBPD) was the only up-regulated transcription factor in these two brain regions of all three models. Network analyses revealed that CEBPD has functional relationships with genes in transcription, pro-inflammation, cell death, binding, myelin and transport.

**Conclusion:**

These results show that: 1) Regionally specific gene expression abnormalities precede the brain histological and neuronal function changes, 2) Temporal gene expression profiles provide insights into the molecular mechanism during the GSL storage disease course, and 3) CEBPD is a candidate regulator of brain disease in prosaposin deficiency to participate in modulating disease acceleration or progression.

## Background

The physiological importance of prosaposin has been demonstrated by the genetic deficiencies of individual saposins or prosaposin that lead to various glycosphingolipid (GSL) storage diseases [1-4]. Saposin B deficiency leads to sulfatide accumulation and a metachromatic leukodystrophy-like disease [4] that is similar to the deficiency of arylsulfatase A, its cognate enzyme. Saposin C activates acid β-glucosidase and its deficiency leads to a Gaucher-like disease with an excess accumulation of glucosylceramide in cells [2]. Saposin A deficiency in mice results in a late onset, chronic form of globoid cell leukodystrophy [5], whereas deficiency of saposin D in mice causes a loss of Purkinje cells and urinary system defects [6]. The critical roles for saposins in GSL metabolism are highlighted by the extensive GSL storage in various central nervous system (CNS) regions in the human and mouse prosaposin deficiencies [1,3]. This deficiency leads to gross abnormalities in CNS degradation of lactosylceramide (LacCer), glucosylceramide (GC), sulfatide and galactosylceramide with consequent pathologic accumulation of these GSLs and gangliosides.

Targeted disruption of prosaposin in the mouse leads to a complex neurodegeneration with neuronal and microglial accumulation of GSLs, and demyelination [1]. The excesses of GSLs lead to neuronal losses with regional specificity and death by ~30 days [1]. Our hypomorphic prosaposin model PSKO-TG (PS-NA) containing a prosaposin transgene has 45% of saposin protein expression in brain, survives up to 220 days and has delayed onset of neuropathological changes and Purkinje cell loss compared to the null mouse [7]. Another mutant mouse, 4L/PS-NA, has the acid β-glucosidase V394L/V394L (4L) point mutation combined with hypomorphic expression of the prosaposin transgene (PS-NA) [8]. This mouse shows accumulation of GC in visceral organs and the CNS in excess of that in either 4L [9] or PS-NA mice. Similar to PS-NA mice, 4L/PS-NA mice develop a neurological phenotype and loss of Purkinje cells. Neuronal GSL storage and activated microglia/macrophage cells and astrocytes in CNS are common pathologies in all three models.

Here, the temporal course of the neuronal phenotypes was correlated with the molecular profile of disease progression in these prosaposin deficiency mouse models. Microarray analyses revealed the common transcription factors that underlie prosaposin pathology and their relationship to development of the neurological phenotype. The results provide insights into the molecular mechanisms and the potential for strategic interventions for this class of diseases, as well as other acquired CNS degenerative disorders that involve GSL.

## Results

### Description of prosaposin deficient mouse models

Three mouse models with prosaposin deficiencies are included in this study (Table 1). Prosaposin knock out mice (PS-/-) live about 30 days and have progressive accumulation of LacCer, GC, ceramide and total ganglioside sialic acid in the CNS [1]. Neuronal GSL storage is evident in PS-/- newborn brains [10]. PS-/- mice show the onset of neurological signs at ~20 days of age and the phenotype rapidly progresses during the next 5–10 days. PS-NA mice have ~45% of normal levels of prosaposin protein expression in the brain and lesser levels in other tissues [7]. They survive up to 32 wks with slow progression of neurological deficits. LacCer and GC are the predominant excess neutral GSLs in the PS-NA brains. PS-NA mice produced normal size litters and this model was subjected to neurological and behavioral testing. 4L/PS-NA mice are homozygous for a *gba *mutant gene encoding a V394L GCase against the PS-NA background [8]. The phenotype includes an average life span ~22 wks, progressive neurological deficits, and greater excesses of GC accumulation in brain and visceral tissues than those in PS-NA mice. PS-/- mice did not reproduce and 4L/PS-NA had reduced fertility. PS-NA and PS-/- mice were in the FVB strain background. 4L/PS-NA mice were in a mix strain background (50% FVB, 25% C57BL/6, 25% 129SvEvBrd). WT mice in the FVB background were used as controls in this study.

**Table 1:** Prosaposin deficient mouse models

**Genotype**	**Strain (%)**	**Life Span**	**Major CNS lipids (Accumulation level)**	**CNS phenotype**	**Prosaposin level in CNS (% WT)**	**References**
PS-/-	FVB	~4 wks	14 – 30 day GC (+ → +++) LacCer (+ → +++)	Ataxia, Tonic status epileptics	0	[1,10]
PS-NA	FVB	32 wks	4 – 22 wk GC (- → +) LacCer (- → +)	Ataxia, waddling gait	45	[7]
4L/PS-NA	FVB (50) C57BL/6 (25) 129SvEvBrd (25)	22 wks	4 – 20 wk GC (+ → +++) LacCer (- → +)	Ataxia, waddling gait	45	[8]

### Impairment of neurological function

**Figure 1:**
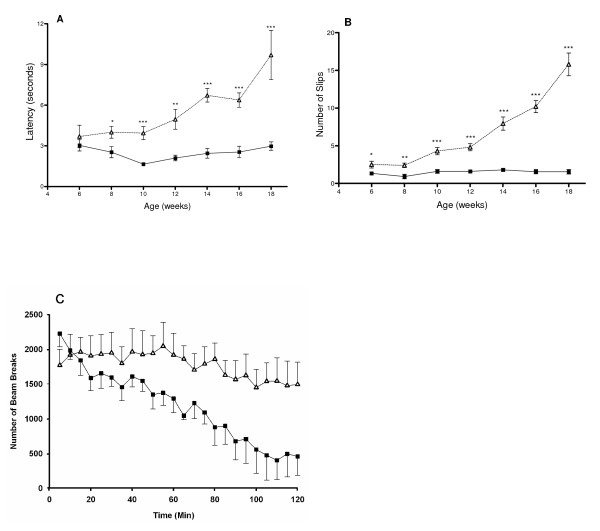
**Progressive neurological deficits**. (A, B) Narrow bridges. Mice were trained for three days. The latency to cross the bridge (A) and the number of foot slips (B) on a round wood beam (11 mm) were recorded (PS-NA, n = 10; WT, n = 10). PS-NA mice [open triangles] made significantly more foot slips and slower latency than WT [black squares] (*p < 0.05, **p < 0.01, ***p < 0.001). (C) Locomotor test at 18 wks. The mice were placed in an activity chamber for 120 min and locomotor activity was recorded in 5 min intervals (PS-NA, n = 5; WT, n = 5). PS-NA [open triangles] mice showed progressive failure to habituate compared to WT [black squares] mice.

Deterioration of neurological function was formally assessed in the PS-NA mice. The narrow bridges test was performed to evaluate balance and motor performance (Fig. 1). Different levels of task difficulty were assessed with round- and square-shaped beams with varying cross-sectional areas. At 6 wks, control and PS-NA mice walked along the beam with similar latencies (Fig. 1A). However, PS-NA mice made significantly more foot slips than controls on medium and narrow beams (Fig. 1B). At 10 wks, PS-NA mice were already significantly slower in traversing the narrowest round and square beams, and the number of foot slips increased on medium and small square beams. Between 12 to 18 wks, PS-NA mice showed increased difficulty in traversing all beams, as measured by their increased latency and foot slips compared with control mice (Fig. 1A and 1B). At 8 wks or younger, both control and PS-NA mice walked on the beam with an upright posture, but by 12 wks of age, PS-NA mice displayed ventral recumbence; their abdomen was flattened on the surface of the beam, their hind- and fore-limbs were wrapped around the beam, and the fore-limbs were used to drag themselves along the beams. By 14 wks, some mutant mice failed to maintain balance and started to fall, but used their forelimbs to regain balance. PS-NA mice exhibited progressive deterioration in performance on the narrow bridges test until 22 wks.

Locomotor activity is a test of general exploration and response to novelty. PS-NA and WT control mice took > 120 min for adaptation. PS-NA mice showed measurable motor differences starting at 10 wks and the effect was more pronounced by 22 wks. They demonstrated hyperactivity with increases of beam breaks (Fig. 1C), and were active longer than controls. No difference between center and peripheral activity was observed. These results suggest that the deficiency of prosaposin results in a progressive failure to habituate in PS-NA mice, compared to WT mice that showed habituation over the test period. The altered motor function was significant beginning at 12 wks.

Gait was analyzed by footprint patterns while the mice walked through a runway. Between control and PS-NA mice, no significant differences were found before 21 wks for stride length or fore- and hind-paw overlap. By 21 wks, PS-NA mice exhibited significantly increased hind-paw base width from the controls (data not shown).

### Brain neuropathology in prosaposin deficient mice

**Figure 2:**
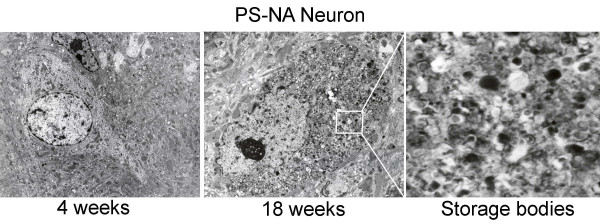
**Electron microscopy showing progressive accumulation of inclusions in neurons of PS-NA brain**. No inclusions were found in neurons of 4 wk PS-NA (A), whereas inclusion bodies were in neuron of PS-NA at 18 wk (B). An enlarged view of inclusion bodies is shown (C). Magnifications are 8,000 X for A and B.

By EM, no inclusions were observed at 4 wks in PS-NA or WT mouse brains. Inclusion bodies were present in neurons of PS-NA mice by 18 wks (Fig. 2). Such inclusions were observed in brain stem, midbrain and cerebral cortex, but rarely in Purkinje cells. 4L/PS-NA mice had neuronal storage similar to that in PS-NA brains. However, 4L/PS-NA mice showed large amounts of storage in granular cells of the cerebellum; this is not observed in PS-NA mice [8]. PS-/- mice show inclusions in neurons of various regions in the brain [10].

**Figure 3:**
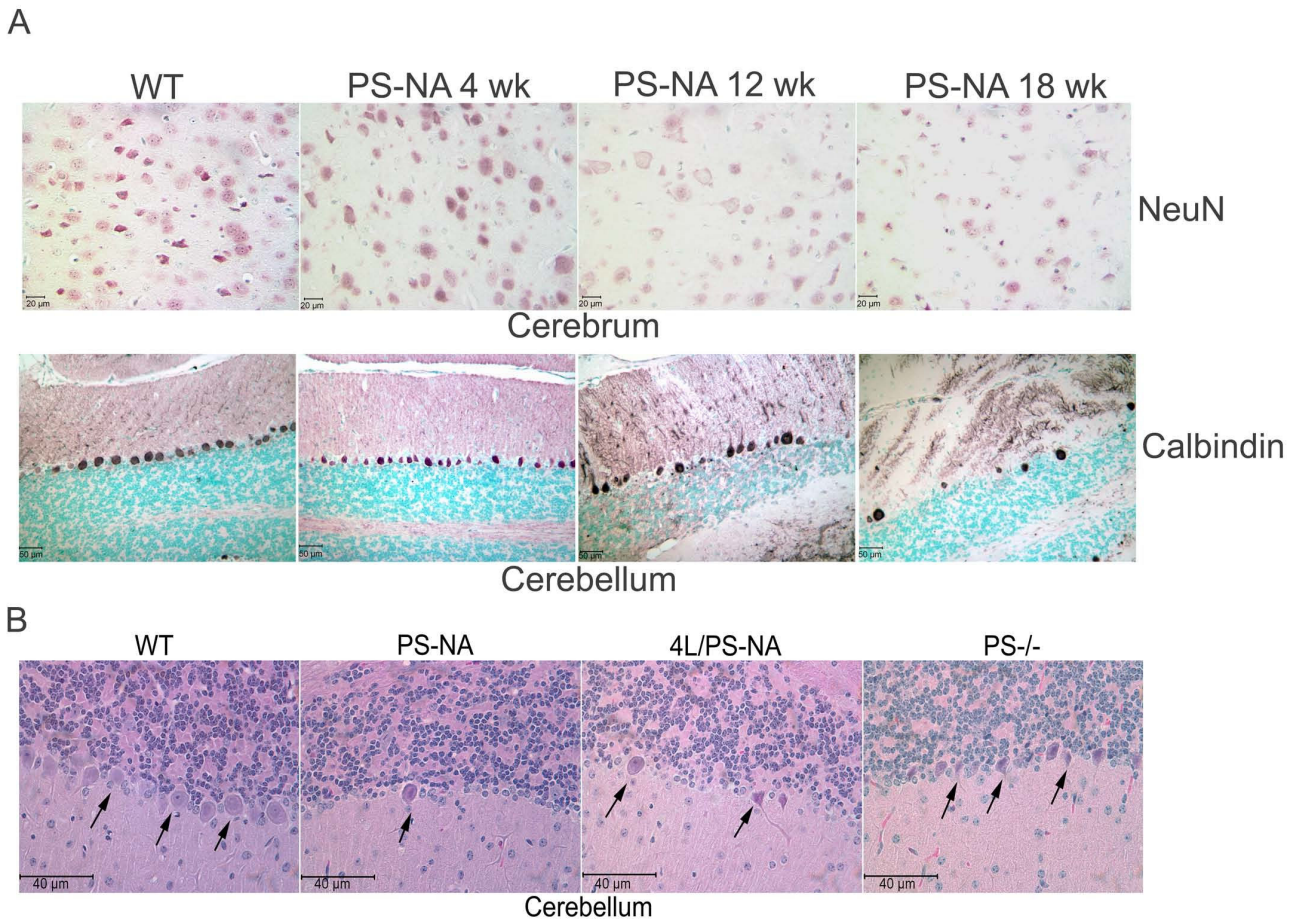
**Neuronal degeneration in brain**. (A) Paraffin sections were stained with a neuron marker, NeuN, and a Purkinje cell marker, Calbindin D28K (calbindin). Progressive decreases in NeuN stained cells (upper panel) and incremental loss of Purkinje cells (lower panel) were shown in PS-NA mice at 12 wk and older. The NeuN changes were generalized, but regional variations in neuronal loss also were evident. (B) H&E stained paraffin sections of cerebellum showed the presence of Purkinje cells (arrows) in PS-/- (25 day) and WT (18 wk) mice and loss of Purkinje cells in PS-NA (18 wk) and 4L/PS-NA (18 wk) mice.

Extensive neuronal degeneration was found in all the prosaposin deficient mouse brains. The specific neuronal stain, NeuN, was decreased in the cerebral cortex at 12 and 18 wks in PS-NA mice. Losses of cerebellar Purkinje cells were demonstrated with anti-Calbindin D28K, a Purkinje cell marker, beginning at 12 wks (Fig. 3A). Neuronal degeneration and decreases in Purkinje cell numbers were also observed in 4L/PS-NA mice. PS-/- mice showed neuronal degeneration in thalamus and cortex starting at ~20 days; no Purkinje cell loss was observed (Fig. 3B).

### Microarray analyses of brain tissues in prosaposin deficient mice

To explore the genes involved in the prosaposin deficiency disease progression pathway, mRNA microarray analyses were performed in PS-NA mice using Mouse Genome 430 2.0 Array chips (Affymetrix). Partek^® ^Genomics Suite was used for statistical analyses of the microarray data. The commonalities and specificities of gene expression were analyzed in PS-/-, PS-NA, and 4L/PS-NA mice. PS-/- mice (total PS deficiency) quickly develop the neurological phenotype and have a short life span [1]. 4L/PS-NA mice have the same level of prosaposin expression as PS-NA mice, but the mice have a shorter lifespan, and also have greater GC accumulation (Table 1) [7,8]. The gene profiles were analyzed in two general brain regions, cerebrum and cerebellum, that were collected at several time points for PS-/- (0, 10, 20 and 25 days), PS-NA (0, 4, 12 and 18 wks) and 4L/PS-NA (4, 12, and 18 wks). The probe sets for each gene in the mutant mice that showed significant differential gene expression were compared to the age-matched WT (Table 2). The significantly differentially expressed probe sets in the mutant models ranged from 460 to 2000 of the 45,101 total probes on the chip. For further analyses, the probe sets were replaced by the genes. ESTs and genes of unknown function were excluded. At a false positive rate (FDR) of 0.2, the total differentially expressed genes in each model ranged from 1.0–4.4% of the total genes on the chip. The complete lists of differentially expressed genes, grouped for their functions, are in Additional file 1.

**Table 2:** Summary of differentially expressed probesets and genes in brain regions of prosaposin deficient mouse models

	**PS-/-**		**PS-NA**		**4L/PS-NA**	
	
	Cerebellum	Cerebrum	Cerebellum	Cerebrum	Cerebellum	Cerebrum
Number of differentially expressed probesets	460	468	915	462	1983	635
EST and unknown genes	58	27	112	48	156	62
Differentially expressed probesets with known genes	402	441	803	414	1827	573
Differentially expressed genes	349	367	662	366	1512	493
% of differentially expressed genes on chip	1	1.1	1.9	1.1	4.4	1.5

Compared to WT mice, the total number of significantly differentially expressed genes in each model was in the following order: 4L/PS-NA > PS-NA > PS-/- (Table 2). In PS-NA and 4L/PS-NA mice, the number of differentially expressed genes was greater in cerebellum than in cerebrum. PS-/- mice had about equal numbers of differentially expressed genes in both tissues and ~20% were in common between the cerebrum and cerebellum. Temporal profiling revealed that the number of the differentially expressed genes increased with age in all three models (Table 3). The differentially expressed genes were sub-grouped into those up-regulated (expression level ≥ 1.5 fold) or down-regulated (≤ 1.5 fold) for any time point for each tissue (Table 3). The number of up-regulated genes was nearly constant at the second time point (10 days for PS-/- and 4 wk for PS-NA and 4L/PS-NA), and then increased by the third time point (20 days for PS-/- and 12 wks for PS-NA and 4L/PS-NA). The number of down-regulated genes was relatively constant throughout. Even at birth, ~100–300 genes in cerebellum or cerebrum had differential expression in PS-NA and PS-/- mice (Table 3); 4L/PS-NA mice were not done. Although the PS-NA and PS-/- mice did not develop neurological signs until after 6 wks and 14 days, respectively (Table 1), the alterations of mRNA expression preceded the neuronal deficits. These data suggest that the number of genes involved in pathological progression increased with age and varied with the genotype and lifespan, and the molecular alterations preceded the histological and neurological changes.

**Table 3:** Temporal profile of differentially expressed genes in cerebellum and cerebrum of prosaposin deficient mouse models

**Genotypes**	**Tissues**	**Expression**	**Number of differentially expressed genes**						
			0 day	10 days	20 days	25 days	4 weeks	12 weeks	18 weeks

**PS-/-**	Cerebellum	up	151	97	212	213			
		down	58	43	83	83			
		**subtotal**	**209**	**140**	**295**	**296**			
	Cerebrum	up	134	70	250	288			
		down	22	52	66	64			
		**subtotal**	**156**	**122**	**316**	**352**			

**PS-NA**	Cerebellum	up	134				238	265	390
		down	164				99	87	124
		**subtotal**	**298**				**337**	**352**	**514**
	Cerebrum	up	54				100	212	232
		down	61				59	48	22
		**subtotal**	**115**				**159**	**260**	**254**

**4L/PS-NA**	Cerebellum	up					436	868	831
		down					270	428	313
		**subtotal**					**706**	**1296**	**1144**
	Cerebrum	up					216	347	341
		down					88	98	67
		**subtotal**					**304**	**445**	**408**

**Figure 4:**
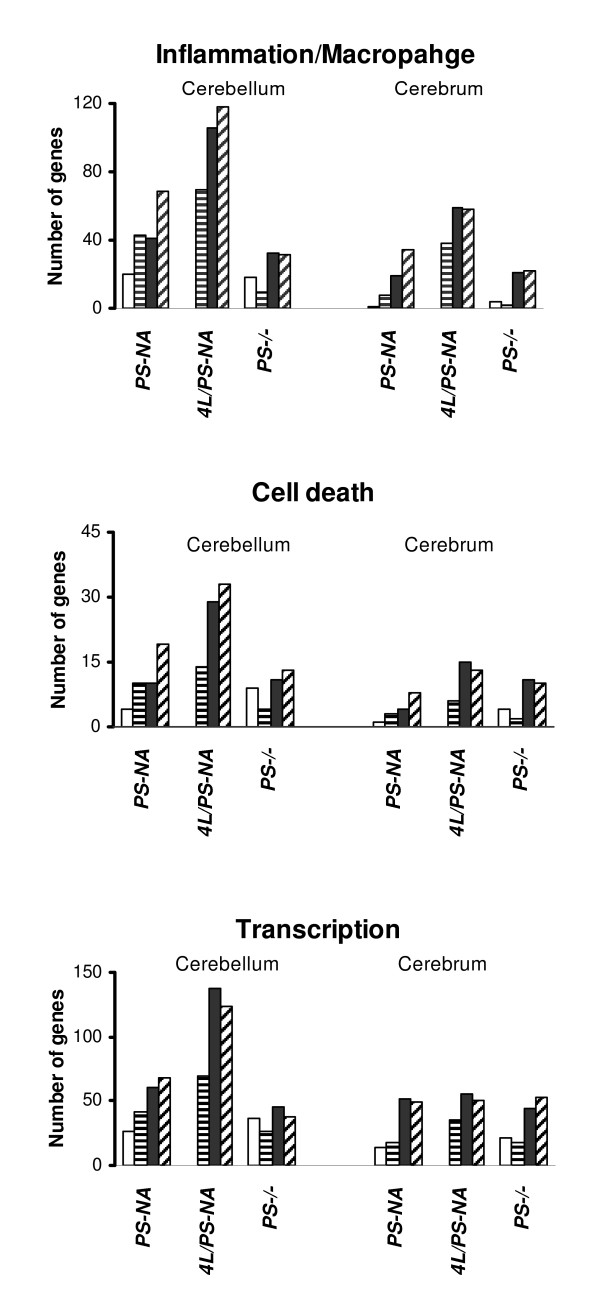
**Temporal expression of three functional categories ascribed to genes determined from the Gene Ontology (GO)**. The number of differentially expressed genes in Inflammation/Macrophage, Cell death and Transcription categories increased with age and were greater in the cerebellum than in the cerebrum. Each column represents the number of differentially expressed genes at that time point. Time points for PS-NA and 4L/PS-NA are 0 day (white columns), 4 wk (line columns), 12 wk (black columns) and 18 wk (hatched columns); for PS-/- are 0 (white columns), 10 (line columns), 20 (black columns) and 25 (hatched columns) days.

Ontological analyses of the differentially regulated genes revealed involvement in a broad spectrum of cellular functions. The functional groups with statistical significance across the cerebrum and cerebellum in the three animal models included neuronal conduction (myelination and synaptic vesicles), cell growth and metabolism, proinflammatory response (inflammation, macrophage, extracellular matrix and cell adhesion molecules), cell death, and signal transduction (Table 4 and Additional file 1). All three mouse models showed similar spectra of gene expression patterns for these functional groups, except cerebra of PS-NA and 4L/PS-NA or cerebrum of PS-NA which did not show significant (p > 0.05) involvement of synaptic or myelination genes, respectively. Several of these categories presented consistent increases of up-regulated genes with age, particularly those in the groups for inflammation and adhesion, cell death, signal transduction, transcription factors and transport (Fig. 4 and Additional file 2). The total numbers of genes in these groups increased with age. More than twice as many genes were up-regulated in cerebellum than cerebrum. It is likely that additional dysregulated genes will be identified as sub-regions of cerebrum are analyzed. 4L/PS-NA mice had twice as many up-regulated genes as the PS-NA mice in cerebellum or cerebrum. This was primarily associated with more rapidly progressive neurological signs in the 4L/PS-NA mice. The mixed strains in 4L/PS-NA might also contribute to the larger numbers of differentially expressed genes, but the complexity of the genotype (5 separate loci) and low number of offspring precluded exact matching of the background strains.

**Table 4:** Functional classification of significantly expressed genes in cerebellum and cerebrum of prosaposin deficient mouse models

	**PS-/-**				**PS-NA**				**4L/PS-NA**			
Categories	Cerebellum		Cerebrum		Cerebellum		Cerebrum		Cerebellum		Cerebrum	

	No.	*p*-value	No.	*p*-value	No.	*p*-value	No.	*p*-value	No.	*p*-value	No.	*p*-value

Inflammation & Macrophage	34	1.10E-09	23	3.75E-04	80	1.59E-14	39	4.80E-12	147	5.53E-13	78	5.01E-13
Myelination	14	3.57E-11	13	8.21E-10	8	2.07E-03	2	*2.33E-01	11	1.19E-02	6	6.75E-03
Synapse vesicle	5	3.23E-02	14	7.75E-07	14	3.87E-04	4	*1.53E-01	35	3.63E-09	6	*8.86E-02
Cell death	13	8.94E-03	11	4.21E-02	20	1.22E-02	10	4.16E-02	50	4.83E-05	19	1.62E-03
Lipid metabolism	19	1.89E-03	22	2.91E-04	28	6.04E-03	15	3.22E-02	68	1.60E-05	31	9.57E-06
ECM/adhesion	18	1.43E-02	28	1.31E-05	55	8.58E-11	23	8.93E-04	107	7.25E-15	36	2.45E-06
Cytoskeleton organization	15	4.43E-02	20	9.32E-03	20	2.06E-02	19	1.55E-02	57	3.16E-02	26	5.79E-03
Endosome/Lysosome	12	1.32E-04	15	2.90E-06	17	1.96E-04	11	7.06E-04	32	2.11E-05	20	7.99E-08
Signal transduction	50	3.50E-03	75	2.83E-09	131	7.66E-14	68	5.37E-07	288	2.24E-14	89	4.82E-08
Transcription	67	1.70E-02	65	3.92E-02	134	7.55E-04	77	2.43E-03	292	1.01E-04	90	2.09E-02
Transport	69	2.20E-04	80	2.94E-06	108	4.10E-03	69	7.34E-04	288	9.01E-11	83	4.46E-03
Cell growth/metabolism	139	4.31E-10	204	3.13E-14	301	3.28E-07	189	9.13E-10	714	1.98E-13	237	2.87E-08
Binding	142	4.54E-06	172	2.96E-12	278	9.53E-12	169	1.94E-11	641	2.45E-15	201	6.83E-08
Catalytic activity	104	2.57E-03	138	2.96E-09	220	2.70E-08	121	2.68E-05	486	1.90E-13	153	9.12E-05
Others	46		25		71		38		171		47	

The functional categories with *p*-value ≤ 0.05 were considered significant by hypergeometric distribution analyses using R 2.6.0 [56].

No., number of genes.

*, not significant (*p *> 0.05).

All three models had altered expression of many cell death associated genes (Fig. 4). The classification of down-regulated genes (Additional file 2) revealed a relatively constant number with age, implying their direct relationship to prosaposin gene deficiency. In the myelination category, PS-/- mice had more down-regulated genes than PS-NA and 4L/PS-NA mice. Transcription, transport and signal transduction had larger number of altered genes in PS-NA and 4L/PS-NA mice. These results reflect differential tissue and cellular responses to prosaposin deficiency and GSL accumulation with age.

**Figure 5:**
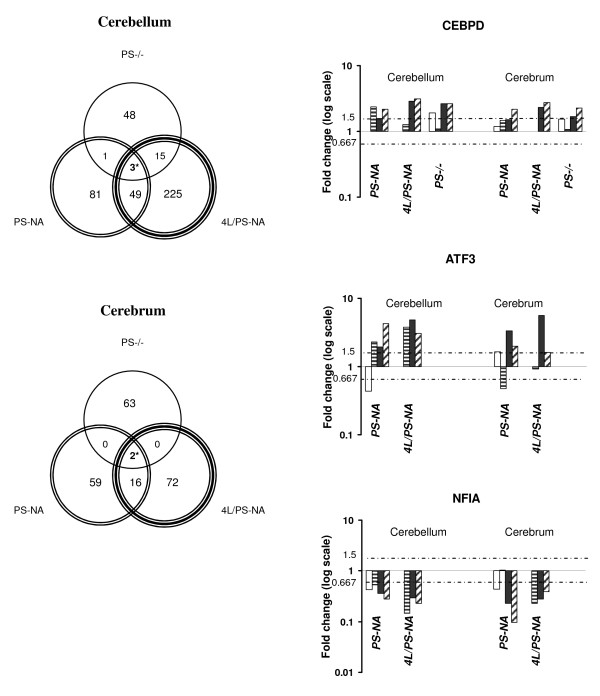
**Regional and temporal expression of transcription genes**. (Left) Venn diagrams of the differentially expressed genes in PS-/-, PS-NA and 4L/PS-NA cerebellum (upper) and cerebrum (lower). The numbers in various regions represent the total of significantly differentially expressed genes in the intersection or complements. For example, 292 genes were significantly differentially expressed in 4L/PS-NA, whereas only 3 genes were shared in PS-/-, PS-NA and 4L/PS-NA cerebellum. Very few genes were shared in three models: Three genes (3*; CEBPD, Efemp1 and Neurod6) in cerebellum and two genes (2*; CEBPD and Man2B1) in cerebrum. Only CEBPD is the common gene in two tissues of three models. There are additional 49 or 16 genes shared by PS-NA or 4L/PS-NA in cerebellum and cerebrum (Atf3, Nfia, etc.). (Right) Expression changes of three shared transcription factors. The expression of CEBPD and Atf3 was increased and Nfia was decreased. Atf3 and Nfia were not altered in PS-/-. Each column presents the fold change at each time point. The dotted lines represent ± 1.5 fold. Time points for PS-NA and 4L/PS-NA are 0 day (white columns), 4 wk (line columns), 12 wk (black columns) and 18 wk (hatch columns); for PS-/- are 0 (white columns), 10 (line columns), 20 (black columns) and 25 (hatch columns) days.

To identify the transcription factors likely involved in disease progression, the commonalities of expression in these three models were analyzed (Fig. 5). As might be expected, the PS-NA gene expression patterns were similar to those found in 4L/PS-NA mice and these two were, in turn, more similar than either alone to those from PS-/- mice. CEBPD was the only transcription factor up-regulated in cerebra and cerebella of all three models; this was observed by 10 days in PS-/- mice, and by 4 wks in PS-NA and 4L/PS-NA mice. CEBPD is a member of CCAAT/enhancer-binding protein family. The expression of CEBPD is found in brain throughout development and into adulthood [11]. The age-dependent expression of CEBPD suggests its participation in the propagation and/or initiation of disease processes in prosaposin deficient mice. Two other transcription factors (Fig. 5), Atf3 and Nfia, were differentially expressed in PS-NA and 4L/PS-NA cerebella and cerebra. Atf3 (activating transcription factor 3) is a member of CREB family of basic leucine zipper transcription factors whose expression begins after birth. In the cerebella of PS-NA and 4L/PS-NA mice, increases of Atf3 expression started at 4 wks and in cerebra at 12 and 18 wks. Atf3 expression was not altered in PS-/- mice during their 4 wk lifespan. The down-regulated transcription factor Nfia (nuclei factor Ia) is a member of CCAAT box binding transcription factor family. Nfia is expressed early in embryonic development to adulthood, and is involved in development of multiple organs, including the hippocampus [12] and cerebellar granular cells [13]. The levels of Nfia were consistently low in cerebrum and cerebellum of PS-NA and 4L/PS-NA mice, but were not altered in PS-/- mice. Atf3 and Nfia could be potential candidates that regulate disease progression in prosaposin deficiency at later ages.

**Figure 6:**
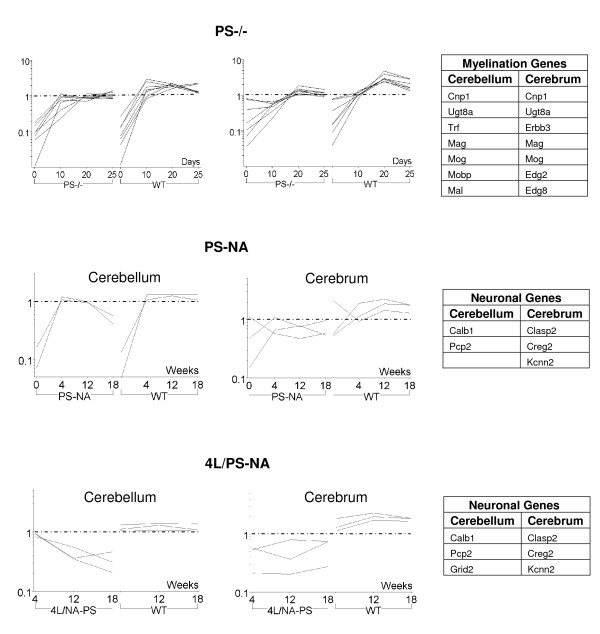
**Down regulated gene expression profiles by K-means clustering analyses**. Upper panels: Expression levels of myelin associated genes were lower in cerebellum and cerebrum of PS-/- mice at 20–25 days compared to WT mice. Middle and Lower panels: Purkinje cell genes, Calbindin (Calb1) and Pcp2, had increase of expression in WT cerebellum with age. Expression of Calb1 and Pcp2 decreased in the cerebellum of PS-NA and 4L/PS-NA mice by 12 wks. Grid2 that also expressed in Purkinje cells was only decreased in 4L/PS-NA cerebellum. In PS-NA and 4L/PS-NA cerebrum, Clasp2, Creg2 and Kcnn2 were decreased compared to WT. Expression profiles for each cluster are shown as the log scale ratio of signal intensity to the median signal intensity across all samples with age (days or weeks). The right side tables show the genes in the clustering.

The down-regulated genes were analyzed by K-means clustering for similarities in expression profiles throughout development (Fig. 6). The myelination-associated genes with similar expression profiles showed decreased expression levels in cerebellum and cerebrum of PS-/- mice by 10 days compared to WT (Fig. 6 and Additional file 2). These genes were not significantly altered in PS-NA and 4L/PS-NA mice. This result is consistent with findings that hypomyelination is a major component of the phenotype in PS-/- mice, but not of PS-NA mice [1,7]. PS-NA and 4L/PS-NA mice showed losses of cerebellar Purkinje cells (Fig. 2). Down-regulation of Calb1 (Calbindin 28 k) and Pcp2 (Purkinje cell protein 2) was detected by 12 wks in cerebella from PS-NA and 4L/PS-NA mice (Fig. 6). Grid 2 is a delta 2 glutamate receptor that controls synaptic plasticity in Purkinje cells [14]. Decreases of Grid 2 were found in the cerebellum from 4L/PS-NA mice suggesting the loss of Purkinje cells was related to synaptic arborization. Overall, PS-NA mice had fewer down-regulated genes than 4L/PS-NA mice (Table 3 and Additional file 2); this was likely associated with the more rapid development of neurological signs in 4L/PS-NA mice than in PS-NA mice, as well as the degree of GC accumulation. In cerebrum, three down-regulated genes were shared in the PS-NA and 4L/PS-NA brains. Creg2, a cellular repressor of the E1A-stimulated gene, is specifically expressed in hippocampus and is involved in cellular differentiation [15]. Kcnn2 (Ca^2+^-activated K^+ ^channel type 2) modulates aspects of hippocampal learning, memory, and synaptic plasticity [16] and is expressed only in the brain. Clasp2 (cytoplasmic linker protein associating protein) binds to microtubule ends and is involved in cytoskeletal organization [17]. Down-regulation of Kcnn2, Creg2 and Clasp2 suggests defects in cellular differentiation and synaptic activity in PS-NA and 4L/PS-NA mouse brains.

**Figure 7:**
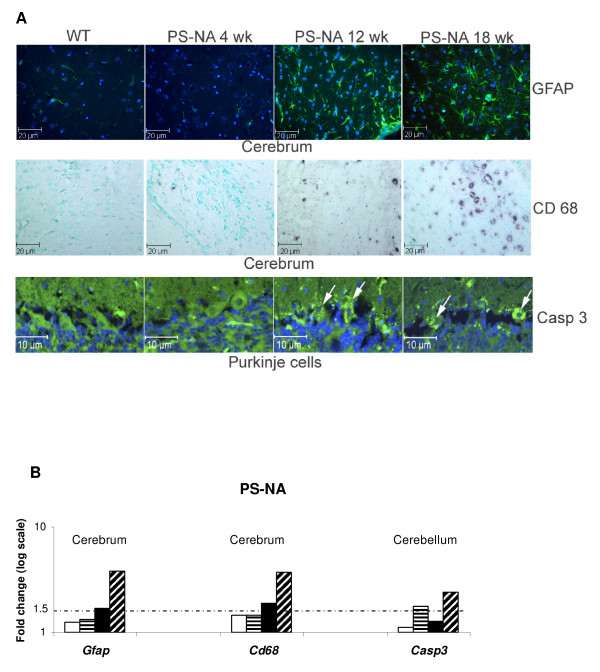
**Immunohistochemical verification of selected genes**. (A) Consistent with microarray (chip) results, GFAP (green fluorescence) showed signal enhancement in astrocytes of cerebrum in 12 and 18 wk PS-NA mice. CD68 (black) microglia/macrophage stains showed sparse positive cells in cerebrum of WT and 4 wk PS-NA mice. CD68 signal increases were evident in 12 and 18 wks PS-NA mice. Caspase 3 (Casp3, green fluorescence) signals stained Purkinje cells (arrows) in PS-NA 12 wk and older mice. DAPI (blue fluorescence) stains nuclei. (B) Microarray (chip) results of expression changes for GFAP, CD68 and Caspase 3. The dotted line represents 1.5 fold. Time points for PS-NA and 4L/PS-NA are 0 day (white columns), 4 wk (line columns), 12 wk (black columns) and 18 wk (hatched columns); for PS-/- are 0 (white columns), 10 (line columns), 20 (black columns) and 25 (hatched columns) days.

With the disease progression, increasing numbers of proinflammatory associated genes (macrophage genes, cytokines, chemokines, complemented molecules, CD molecules, ECM and adhesion molecules, MHC) showed increased mRNA levels in brains of all three models (Additional file 1) and included the macrophage expressed genes: Csf, Ccl3, CD68, C1q, Lgals3. GFAP level increases indicated astrogliosis in the PS deficiency diseases (Additional file 1 and Fig. 7). The expression levels of proinflammatory associated genes were up-regulated by 4 wks and before neurological impairment. Several extracellular matrix and proteolytic enzymes (Mmp12, Timps, procollagen integrin b2) and lysosomal proteases (cathepsins) involved in the proinflammatory response also had increased expression levels. The cell death genes, Pycard and Bcl2a1a, had elevated expression in cerebella and cerebra from all three models. Pycard and Bcl2a1a are apoptotic protease activator and anti apoptosis molecules, respectively [18]. Casp3 was up-regulated in the cerebellum from PS-NA mice, but not that from PS-/- mice (Additional file 1 and Fig. 7), suggesting tissue and age-dependent activation patterns. In addition, some of the genes involved postsynaptic activity (synaptotagmin and dynamin) and proteasome subunit proteins (Psm) and Ubiquitin associated enzymes (Usp and Uchl)) were significantly up-regulated in the PS-NA, 4L/PS-NA and PS-/- models (Additional file 1), implicating altered synaptic activity and ubiquitin proteasome metabolism in prosaposin deficient mice secondary to GSL accumulation in brain.

### Validation of microarray data

**Figure 8:**
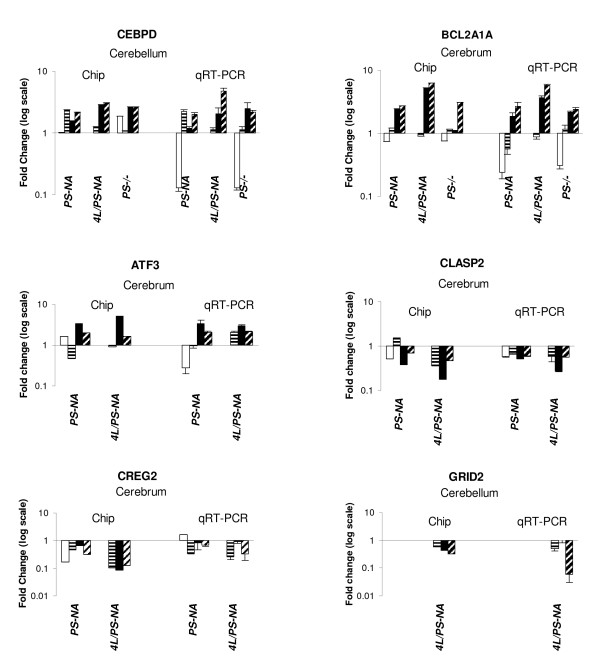
**Quantitative RT-PCR verification of selected gene expression levels**. The gene expression changes were normalized to actin signals and presented as the fold changes for each gene relative to age-matched WT samples. For each bar graph, the left is the microarray (chip) results and the right is qRT-PCR results (n = 3). The changes in expression levels from both analyses trended similarly. (Time points for graph are labeled as in Figure 7).

To validate the expression changes identified in the array analyses, selected genes were examined in more detail by qRT-PCR. The alterations of gene expression for CEBPD, Atf3, Creg2, Clasp2, Grid 2 and Bcl2a1a directly corresponded with the specific microarray data, e.g. increases of mRNA for CEBPD, Atf3 and Bcl2a1a, and decreases for Greg2 mRNA were found in the adult samples (Fig. 8). By immunofluorescence analyses, GFAP signals in 4 wk PS-NA brain was at WT levels. In comparison, the GFAP signals in astrocytes of 12 and 18 wk PS-NA mice were greater. In addition, GFAP-positive astrocytes in PS-NA mice were increased in number and were ramified across brain regions compared to control samples (Fig. 7A). Such astrogliosis is a hallmark of many neurodegenerative diseases. A few CD68 (macrophage marker) positive cells were present in 4 wk PS-NA brain. In 12 and 18 wk PS-NA mice, CD68 stained microglia/macrophages in all regions of the brain, but with increased intensities. Some of these CD68-positive cells had increased size. Caspase 3 showed elevated expression in Purkinje cells of the cerebellum in 12 and 18 wk brains. GFAP and CD68-positive staining were also observed (in a similar distribution) in PS-/- and 4L/PS-NA mice (data not shown). These results were consistent with the microarray analyses (Fig. 7B) and demonstrated that astrocytes and microglia were activated in these models and involved in the proinflammatory response in an age-dependent manner.

### CEBPD associated pathway

**Figure 9:**
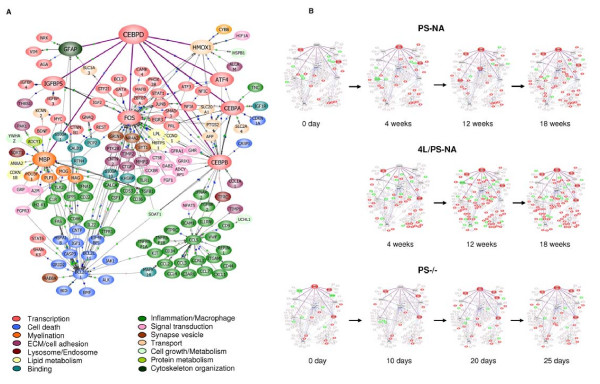
**The CEBPD network**. (A) The differentially expressed genes from three models were constructed into an interaction network associated with CEBPD as a master regulated gene in PathwayArchitect. 152 genes with a high confidence index of direct and indirect interactions with CEBPD were included in the pathway. Connecting lines between gene symbols indicate interactions, the purple thick solid lines indicate the direct interactions between CEBPD and 7 genes, and they are GFAP, MBP, IGFBP5, FOS, CEBPB, CEBPA, ATF4 and HMOX1. Each of those genes connects to multiple functional genes highlighted with different color. Different types of interactions are denoted by symbols on the lines. Links scheme: Green square indicates regulation; arrow, direction of regulation; blue square with black border, binding; blue square, expression; orange circle, protein modification; green diamond with blue border, metabolism; green circle, promoter binding; light green triangle, transport; + in gray circle, positive effect; and – in gray circle, negative effect. (B) Temporal profile of CEBPD network in cerebellum of three models. Red nodes show gene expression level increase. Green nodes show gene expression decrease. Grey nodes present no significant change.

The differentially expressed genes in the three models were subjected to network-based analyses to explore CEBPD gene interactions. About 152 genes were classified into a CEBPD- associated common pathway from all differentially expressed genes in three models. CEBPD had direct interactions with GFAP, MBP, IGFBP5, ATF4, HMOX1, CEBPA, CEBPB and FOS. Those genes in turn connected with those in multiple functional groups: transcription (ATF3 and NFIA), proinflammation (CCL3, TLR2 and CSF1), cell death (CASP3 and BCL2L), binding (CALB1 and PCP2), myelin (MBP, PLP1 and MOG) and transport (KCCN2) (Fig. 9A). The temporal expression pattern (Fig. 9B) in the cerebellum from each model showed that up-regulated genes in the CEBPD network increased and down-regulated genes were constant with time. The pathway does not reflect cellular specificity, but it provides a temporal view of dynamic changes for each functional group in the disease course.

## Discussion

Deficiency of prosaposin/saposins results in incomplete catabolism of GSLs and their age dependent accumulation in the brain [7,8]. To understand the molecular changes at the transcriptome level during the disease course of prosaposin deficiency, three mouse models were compared in this study by microarray analysis: 1) a complete deficiency [PS-/-], 2) a hypomorph [PS-NA], and 3) a hypomorph together with a GCase mutation [4L/PS-NA]. In these three chronic neurological disease models, significant numbers of genes were up- or down-regulated at birth prior to any behavioral, histological or significant GSL abnormalities. The differentially expressed genes included those involved in transcription and signal transduction that were early molecular responses to the insult from incomplete GSL degradation. With age, GSL (Table 1) accumulation became detectible and the surrounding astrocytes and microglial cells sensed the insult and promoted proinflammatory responses to maintain tissues homeostasis. The alterations of proinflammatory genes were common in all three models, which reflect the generalized distribution of microglial cells and astrocytes in the brain. Prolonged proinflammatory reactions also are present in other neurodegenerative diseases [19,20]; they can promote beneficial effects, i.e., repair, as well as detrimental or destructive effects in the brain. The loss of irreplaceable neurons in PS-NA and 4L/PS-NA mice started at ~12 wks. The negative TUNEL assays [7] indicated an alternative cell death pathway, e.g., necrosis or autophagy, in these mouse brains. Importantly, neurobehavioral impairments in PS-NA and 4L/PS-NA mice preceded detectible neuronal cell loss, suggesting that the functional impairments preceded the structural deficits, and that the accumulating (even subclinical) GSL had direct effects on overall cell regulation. The basis for the underlying neurological deficits might be attributed to synaptic dysfunction as has been found in ataxia and Alzheimer's disease [21,22]. Alteration of synaptic and proteosome genes in the current models implicates GSL storage in the lysosome as affecting synaptic function and leading to dendritic retraction and neuronal death. As a potential link to this pathogenic mechanism, ubiquitin proteases that are linked to synaptic activity [22,23] were altered in these models. Differential expression of the ubiquitin protease genes was identified in PS-/-, PS-NA and 4L/PS-NA mouse brains and accumulation of ubiquitin was found in the brains of all three mouse models (Sun, et al., unpublished observation). Clarification is needed for the interaction between ubiquitin-dependent protein turnover, and the plastic and degenerative changes at synapses in brains with GSL accumulation.

Among the differentially expressed genes, CEBPD was the only transcription factor up-regulated in the cerebella and cerebra of all three mouse models. This unique commonality was not obscured by the potential diluting effect from the combined analyses of sub-regions of cerebrum or genetic background variation between 4L/PS-NA (FVB/C57BL/129) and PS-/-, PS-NA and WT (FVB). Network analysis indicated that CEBPD directly or indirectly connects to gene functional effector pathways. For example, CEBPD modulates GFAP and expression of numerous cytokines through FOS mediated proinflammatory pathways (Fig. 9). CEBPD also modulates MBP and FOS expression. MBP interacted with myelin-associated genes and both MBP and FOS connected into the cell death pathways. CEBPD has been previously implicated in peripheral immune challenge [24] and CEBPD is up-regulated after traumatic brain injury in rats and in brains from patients with Alzheimer's disease [25,26]. Lipopolysaccharide (LPS) and galactosylsphingosine are exogenous ligands that regulate CEBPD expression in visceral tissues and astrocytes [27-31]. In response to LPS treatment, CEBPD and NF-kB are recruited to the CNS [32]. Galactosylsphingosine, a cytotoxic metabolite of galactosyl ceramide found in Krabbe disease, induces expression of cytokine mediated nuclear translocation of CEBPD [28]. The present data suggest that accumulated GSLs in prosaposin deficient brains might be endogenous lipids that mediate CEBPD expression. Furthermore, CEBPD binds to cytokine promoters in macrophages and astrocytes [33,34]. The expression of CEBPD is mediated by Sp1, STAT3, c-Rel and c-Jun [35,36], whereas the downstream genes MBP and GFAP, as well as several inflammatory genes, are modulated by CEBPD in astrocytes [30,37].

CEBPD is widely expressed in the PNS and CNS. CEBPD in combination with CREB (cyclic AMP-responsive element-binding protein) participates in nerve growth factor gene transcription [38]. Curiously, the CEBPD knockout mice have no major phenotypes except enhanced contextual fear, whereas, CEBPA and CEBPB knockouts exhibit perinatal lethality or rapid deterioration within months after birth [39-41]. Such findings suggest that CEBPD is not essential in non-stressed physiological development, but may be important in response to pathological insults by playing a role in the modulation of proinflammatory responses and neuronal homeostasis in neurodegenerative diseases. CEBPD expression was up-regulated after 10 days in PS-/- mice and after 4 wks in PS-NA and 4L/PS-NA mice. The temporal change of this expression correlated directly with the biochemical and histological progression of the disease. Apparently, CEBPD plays a role in the CNS of prosaposin deficiency mice in promoting disease progression.

Atf3 has been suggested as an "adaptive response" gene by responding to extra- and intra-cellular changes induced by stress signals and signals for promoting cell proliferation [42]. Atf3 is induced by LPS and is a negative regulator of TLR 4 and Ccl4 released from macrophages [43,44]. Atf3 has been suggested as having a role in controlling gene expression programs required for axon regeneration [45]. The increase of Atf3 might be involved in an adaptive response to the disease in the present models. Nfia promotes gliogenesis in spinal cord [46] and directs the differentiation of cerebellar granular neurons [47]. Disruption of Nfia in mice leads to perinatal lethality, agenesis of the corpus callosum, and abnormal formation of midline glial structures [12,48]. Both Atf3 and Nfia were present in the CEBPD network, suggesting potential interactions effects between these transcription factors.

Gene expression profiles have been studied in several lysosomal diseases. By serial analysis of gene expression (SAGE), many up-regulated genes are attributed to the proinflammation pathways in the GM_2 _gangliosidoses, Tay-Sachs and Sandhoff disease patients' brains [49]. In the severe neuronopathic Gaucher disease type 2, with increased GC, the genes in brain associated with microglial cells and astrocytes are slightly elevated and the neuronal genes has small depressions [50]. Using a cDNA microarray, human Niemann-Pick C1 fibroblasts revealed oxidative stress, impairment of trafficking and regulation of calcium in response to abnormal cholesterol and GSL accumulation [51]. This is quite different from that obtained in our prosaposin deficient mice. The transcription profiles demonstrated that molecular changes involved proinflammation, trafficking, and loss of neuron cells. These results suggest a GSL and cellular specificity in response to the disease. Interestingly, several lysosomal enzymes showed up-regulation (β-glucuronidase, α-mannosidase and β-hexosaminidase A and B) in the present models, suggesting altered lysosomal function compensating for GSL storage.

## Conclusion

The microarray analyses in this study begin to dissect the complex interactions that underlie GSL disease progression. Regionally-specific gene expression abnormalities preceded the histological and neuronal phenotype changes. The proinflammatory response of microglial cells and astrocytes likely propagated the disease and played an important role in neurodegeneration. CEBPD is apparently a candidate regulator participated in disease progression in prosaposin deficiency. These results provide insight into the molecular mechanism in the progression of GSL disease and therapeutic potential for the acquired CNS degenerative disorders that involve GSL.

## Methods

### Behavioral assessments

#### Narrow bridges

The mice were tested on square and round wood beams during the training and test phases. Square beams (1 m in length) with cross sectional areas of 25 mm^2^, 12 mm^2^, and 5 mm^2^, and round beams (1 m in length) with diameters of 28, 17, and 11 mm were used. Beams were placed horizontally 50 cm above the floor surface. Both ends of the beam were mounted to narrow supports. The starting point of the beam was illuminated with a 125 W floodlight and two Halogen lights. The end of the beam was attached to an enclosed 20 cm^2 ^dark box. For the training phase, the mice traversed the 12 mm^2 ^beam for 3 consecutive days, 4 trials per day. The first trial was a maximum of 2 min and each subsequent trial was a maximum of 1 min. A successful beam cross was recorded when an animal passed the second support closest to the goal box. Animals were considered to be trained when they crossed the 12 mm^2 ^beam in less than 20 sec. If a mouse failed to traverse the beam on the first day, on the second day the mouse was allowed to start the trials in the middle of the beam. Once the mouse successively traversed the beam, the start position was moved back to the original location. The test phase began on the fourth day and each mouse in turn received two consecutive trials (up to 60 sec/trial) on each of the square and round beams, progressing from widest to narrowest. Latency to traverse each beam and the number of times the hind feet slipped off the beam were recorded. A total of 10 PS-NA and 10 age matched WT mice were included in the test. The tests were started when mice were 6 wks old and repeated every two wks. Student's *t*-test was used to evaluate the data.

#### Locomotor activity

Individual mice (5 PS-NA and 5 WT) were placed in activity chambers for 120 min under standard fluorescent room lighting. The mutant and control mice were tested at the same time in identical chambers, locomotor activity was measured in a 41 × 41 × 30 cm Accuscan activity monitor equipped with 16 pairs of photodetector-LED beams along the x and y axes (Accuscan Electronics with VersaMax software, Columbus, OH). The apparatus was cleaned with 70% ethanol solution between animals. Horizontal activity was recorded in 5 min intervals. PS-NA and WT mice were tested in an alternating order in each test to minimize time of day effects. The test was initiated at 6 wks of age and repeated every three wks.

#### Gait Analysis

Fore- and hind-paws of mice were coated with non-toxic blue and red water-based paint, respectively, and allowed to walk through a 60 cm runway. Footprint patterns were recorded on white paper lining the runway. Stride length and fore- or hind-paw base width were measured.

### Histological Studies

Karnovsky's fixative was used for Electron Microscopy (EM) analyses. For Calbindin D28K staining, paraffin-embedded brain tissue was incubated with mouse anti-calbindin antibody (1/500) (Sigma, C9848). For CD68 and NeuN staining, the paraformaldehyde-fixed tissue sections were used with mouse anti-rat CD68 (Serotec, FA-11) or anti-NeuN antibodies (1/500) (Chemicon, MAB 377). Detection was performed using ABC Vectastain and Alkaline phosphatase kit II-Black (Vector Laboratory). The slides were counter stained with methylgreen (Vector Laboratory) and visualized by light microscopy. For GFAP and Caspase-3 staining, paraformaldehyde-fixed (4%) tissue sections were blocked in 20% goat serum (GS), and incubated in mouse anti-GFAP monoclonal antibody (Sigma, G3893) or rabbit anti-caspase-3 antibodies (Chemicon, AB3623) (1/50 diluted in PBS with 20% GS). FITC conjugated goat anti-mouse or anti-rabbit antibody (ICN/CAPPEL) (1/1000 in PBS) was applied to the sections. The sections were counterstained with Antifade with DAPI (Vector Laboratory). The signals were visualized with a fluorescent microscope equipped with an apotome.

### RNA preparation and Microarray hybridization

Cerebrum and cerebellum were collected from Prosaposin null (PS-/-) mice at 0, 10, 20 and 25 days, prosaposin hypomorphic (PS-NA) at 0, 4, 12 and 18 wks, V394L/V394L glucocerebrosidase mutation and PS-NA mice (4L/PS-NA) at 4, 12 and 18 wks, and WT mice at 0, 10, 20 days and 4, 12, and 18 wks. The strain background of PS-NA, PS-/- and WT was FVB. 4L/PS-NA mice were in mixed strains (50% FVB, 25% C57BL/6, 25% 129SvEvBrd). For each genotype six mice at each time point were used. Total RNA was extracted using TOTALLY RNA kit (Ambion Inc.). RNAs for microarray analysis were generated from six mice at each time point for each tissue, three of which were pooled to make two separate samples for each genotype, brain region, and age. A total of 68 pooled RNA samples (16 for PS-/-, 16 for PS-NA, 12 for 4L/PS-NA and 24 for WT) were submitted to the CCHRF Affymetrix Microarray Core for hybridization to Affymetrix GeneChip Mouse Genome 430 2.0 Array. Labeled cRNA synthesis, GeneChip hybridization, washing and staining followed standard Affymetrix protocols. The probe arrays were scanned by the Affymetrix GeneChip^® ^Scanner 3000. The intensity for each feature of the array was captured with GeneChip Operating Software (GCOS) v1.1.1, according to standard Affymetrix procedures.

### GeneChip quality assessment

Array data was assessed for quality using Affymetrix manual procedures based on scaling factor, 10–50; percent present, > 30%; and housekeeping gene yielding 3'/5' signal ratios, < 3. Global normalization for outliers or "bad chips" was conducted with each sample/chip. The normalized intensity values were subjected to hierarchical clustering to determine the relative similarity of tissues derived from different developmental stages and to identify the outlier(s) or bad chips. Genechips that passed these screenings were used for subsequent analyses to identify genes significantly regulated by prosaposin, time, and region.

### Microarray data

The unprocessed raw microarray data for this study is available at Gene Expression Omnibus database (GEO) at NCBI (accession number: GSE8307).

### Microarray data normalization and analysis

The data for each mouse model and corresponding WT controls, including those from cerebellum and cerebrum, and from 3 or 4 time points were loaded into Partek Genomics Suite 6.2 (Partek Inc., St. Louis, MO, USA) for analyses and normalization using the RMA (Robust Multiarray Average) algorithm [52]. Sample relationships were examined using principal components analysis to reveal any technical effects that would encumber the subsequent analysis. To identify expression changes between genotypes, a mixed-model ANOVA was performed and chosen to partition subject, tissue, age, and mouse genotype. The following linear mixed model (equation) was used to detect differential expression on a gene-by-gene basis:

Y_ijkm _= G_i_+T_j_+A_k_+GTA_ijk_+S_m_+ε_ijkm_

Where y_ijkm _is the expression of the gene for ith genotype, jth tissue, kth age and mth subject. The symbols G, T, A, GTA and S represent effects due to genotype (G), tissue (T), age (A), genotype-by-tissue-by-age interaction (GTA), and subject (S), respectively. The error for each gene for sample ijk is designated as ε_ijkm_. Genotype, tissue and age are fixed effects and subject is a random effect in the mixed model. For each comparison, a linear contrast was set up to obtain the relative fold changes between each mutant to WT control for each tissue at all time points. Six contrasts were added in the computation: PS-/- vs. Control, PS-NA vs. Control and 4L/PS-NA vs. Control in cerebellum and cerebrum, respectively. False Discovery Rate (FDR) was performed to further protect against false positives because of multiple testing [53]. FDR was set at 0.2 and fold change was set at 1.5. Using these criteria, significantly differentially expressed genes were identified in cerebellum and cerebrum for each model through the time course (Table 2). Those genes were sorted into those that were up- or down-regulated (fold change ≥ |± 1.5|) for each time point (Table 3).

### Functional classification

Significantly differentially expressed genes were subjected to an intensive search to identify biological functions. Functional classifications were performed using the Gene Ontology classification obtained through the NetAffx server [54], Gene Ontology database [55], and public information and/or literature references. The hypergeometric distribution was performed using R 2.6.0 [56] to detect the significant functional categories which were regarded significant with *p*-value < 0.05. Based on 1) high scoring homologies of the corresponding encoded proteins to known proteins using biochemical function and molecular process concepts represented in Gene Ontology and 2) the statistical significance of differentially regulated functional groups across two tissues in the three animal models; those differentially expressed genes were grouped into the following categories (Table 4): inflammation/macrophages, myelination, synaptic vesicle, cell death, lipid metabolism, extracellular matrix (ECM)/cell adhesion, cytoskeleton organization, endosome/lysosome, signal transduction, transcription, transport, cell growth/metabolism, binding (nucleotide binding, protein binding, lipid binding, etc.), catalytic activity and others. Some of the genes were grouped into more than one functional category. A complete gene list with temporal expression changes by functional grouping is in Additional file 1.

### Partitioned clustering of gene expression profiles

K means clustering was applied for partitioned clustering to subdivide functionally classified genes into groups based on similarities in expression profiles for each tissue throughout development. Similarities between expression profiles from different genes were determined using the coefficient of shape applied to log_2 _transformed mean expression values for each genotype at each time point. Gene expression profiles for the clusters were visualized using GeneSpring GX 7.3 (Agilent Technologies, Inc., Santa Clara, CA).

### Network construction

The significantly differentially expressed genes in the cerebellum and cerebrum of PS-/-, PS-NA and 4L/PS-NA were loaded into PathwayArchitect 2.0.1 (Stratagene, La Jolla, CA) and built into a CEBPD-associated interaction network. Some interactions were referenced from Ingenuity Pathways Analysis (Ingenuity Systems, Inc.). Each functional group was highlighted in a different color.

### Quantitative RT-PCR

Total RNA was extracted from tissues of three mice using TOTALLY RNA (Ambion Inc.). Those RNA samples used for Quantitative RT-PCR (qRT-PCR) were from the pooled RNAs subjected for Microarray analysis. Reverse transcription of 10 μg of total RNA for each tissue was carried out using High Capacity cDNA Archive Kit (Applied Biosystems) containing Random Hexamer primers. qRT-PCR was performed as previously described using β-actin as an internal control on ABI Prism 7000 Sequence Detection System [57]. Data were presented as means ± S.E. The primer sequences were as in Additional file 3.

## Abbreviations

CEBPD: CCAAT/enhancer-binding protein delta; CNS: central nervous system; MBP: myelin basic protein; GSL: glycosphingolipid; LacCer: lactosylceramide; GC: glucosylceramide; Calb1: calbindin 28K; Pcp2: Purkinje cell protein 2; Casp3: caspase 3; Nfia: nuclei factor Ia; EM: Electron Microscopy.

## Authors' contributions

YS participated in the study design, carried out coordination, data analyses and manuscript drafting. LJ performed the statistic analyses and manuscript drafting. MTW participated in the neurobehavioral data analyses and manuscript drafting. MZ performed the neurobehavioral tests, carried out the sample collection and RNA processing. HR performed the immunohistochemistry assay. BQ participated in the research design and sample collection. BJA participated in the research design and manuscript drafting. CVV participated in the neurobehavioral data analysis and manuscript drafting. DPW participated in the EM and histology studies, and their interpretations. GAG conceived of the study, and participated in its design, data analyses and the manuscript drafting. All authors read and approved the final manuscript.

## Supplementary Material

Additional file 1 Table s1. Functional classification of significantly expressed genes in prosaposin deficient mouse models. Click here for file

Additional file 2 Table s2. Number of up- and down-regulated genes with age in functional categories. Click here for file

Additional file 3 Table s3. A list of primer sequences used in qRT-PCR. Click here for file
